# Measuring the presence and incidence of cholera in Hindustan: New data from primary sources for the colonial era

**DOI:** 10.1038/s41597-023-02796-7

**Published:** 2024-01-08

**Authors:** Maqsood Aslam, Thomas Baudin, Etienne Farvaque, Reda Marakbi

**Affiliations:** 1 LÉP (Laboratoire d’Économie de Poitiers), Université de Poitiers, France, and School of Economics, Quaid-i-Azam University, Islamabad, Pakistan; 2 Univ. Lille, CNRS, IESEG School of Management, UMR 9221 - LEM - Lille Économie Management, F-59000 Lille, France; 3 CIRANO, Montréal, Québec Canada

**Keywords:** Databases, Economics

## Abstract

We build a new dataset covering 90 years of Cholera spreading in Hindustan from 1814 to 1904. We gather data from a collection of primary sources issued from medical reports. We propose a harmonization procedure to make these data comparable and corresponding to the current borders of India, Pakistan, Nepal, and Bangladesh. Our methodology is corroborated when comparing our newly produced data with other accounts, in particular Roger (1926)’s estimations. It opens the door to research aiming at estimating the effect of the successive waves of Cholera on the economic, social and epidemiological dynamics of the region.

## Background & Summary

“(…) cholera swept through the camp of the British Grand Army with “indescribable violence.” Between November 15 and 20 alone, five thousand men, women, and children died^1^. All military maneuvers ceased, as the camp transformed into a hospital and open-air morgue. An eerie quiet descended, broken only by the groans of the dying. The British kept to their tents, venturing out only to inquire about the state of sick friends, while the Indians bore the biers of their dead to the river in silence. At the height of the epidemic, even these rituals ceased. The victims were thrown into ravines or brought to the English tents and left there—the guilt for their deaths laid ceremonially at the door of the colonizing power. Many Indians blamed the epidemic on the slaughter of a cow to feed the British officers in a nearby grove sacred to Hardaul Lala, the deified ancestor of a local noble family.” (Wood^2^, p. 74).

Cholera is a still prevailing disease (Kotar and Gessler^3^). While high-income countries have almost eradicated it, the disease is still a burden on many low- and middle-income countries, and the link between past and current bursts of the disease and their impact on health and development is still not fully understood. The present study documents the presence and prevalence of cholera in Hindustan during the colonial period, and how the data could be used to further our knowledge of health and economic dynamics impacted by the disease.

The dataset we present covers the period 1814–1904. It is based on the coding of primary sources, essentially military ones, that allow us to trace the presence, and the incidence, of the disease. The database relies on the administrative geographic districts of the colonial era, on different levels of disaggregation. This permits to compare other datasets covering the same period (such as the railways, as in Donaldson^4^, or inequality levels, as in Caruana-Galizia^5^). However, we also offer a conversion to present administrative areas, to facilitate the analysis of current issues (India’s productivity divergence, for example – Rodrik and Subramanian^6^).

The data capture the presence and incidence of cholera in a region that was at an historical turning point, as the British colonial state was less and less accepted in the region (as exemplified in the above quote and in Arnold^7^), and its relevance goes beyond the Indian case, as the relationship between diseases, pandemics, and development is a research area of primordial importance (Bloom *et al*.^8^).

## Methods

The dataset represents the evolution of epidemic cholera in what was formerly called Hindustan. Nowadays, this area is divided between India, Pakistan, Nepal, and Bangladesh. The dataset compiles a collection of maps, medical reports, and documents referring to the disease, starting in 1814 and ending in 1904. The first part of the dataset pertains to 1814 to 1824. The second part covers the period 1825 to 1904. The first part is more detailed than the second one, and allows us to detail the epidemic dynamics in a refined way. It allows plotting the incidence for locals and the military separately, with a fine granularity in time and space. Data are aggregated with a monthly periodicity. The frequency of the second part of the dataset is in years.

### Primary sources, period 1814–1824

**Fig. 1:**
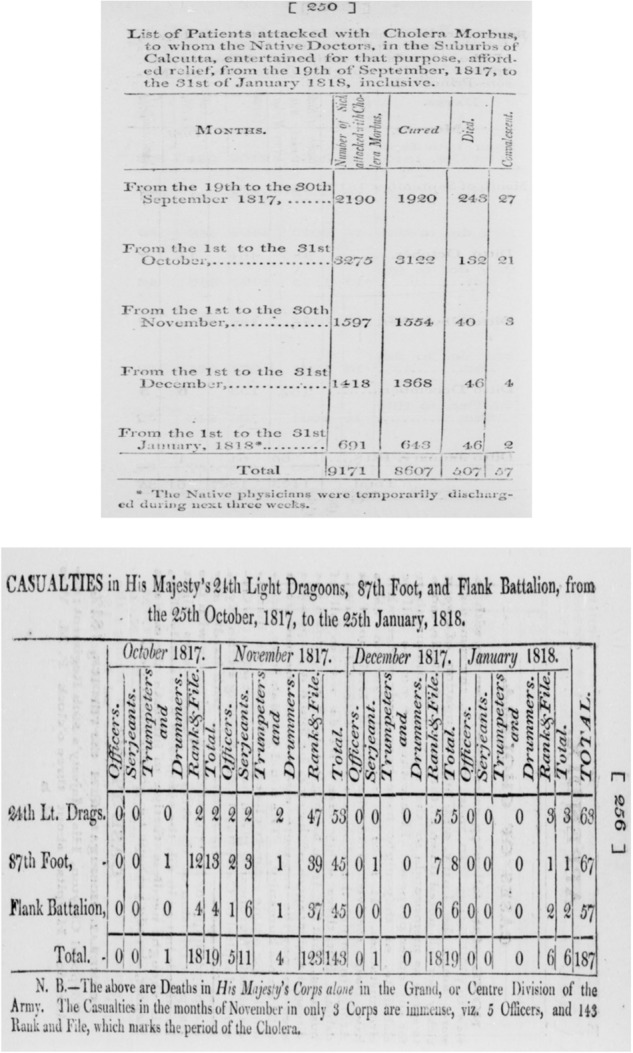
(**a**) Example of primary source – Jameson (1820) – civil victims. (**b**) Example of primary source – Jameson (1820) – military victims.

Most of the parsing effort focused on detailed medical records on cholera in Hindustan in the early 19^th^ century, written by Jameson^9^ and Scot^10^ – see Fig. 1. Both reports are describing concurrent events but Scot’s report puts an emphasis on the passage of cholera in the South and Central India, while Jameson’s focuses on an area extending from the eastern border of Bangladesh to the Pakistani Sindh, its northern border is the southern part of Nepal and reaches central provinces of India. As both reports treat concomitant events, some information overlaps. In the case of overlapping information, the result of Scot’s parsing was selected over Jameson’s, because location names used by Scot offers a more reliable matching between ancient locations and their modern counterparts.

Ancient location names were for the most part difficult to match to current locations and the task would have been even arduous without Hamilton’s geographical description of Hindustan^11^. The hindrance resided in the changing nature of locations’ names across concurrent publications, as appellations remained similar in pronunciation but were different in spelling. One important source of these discrepancies comes from the oral transmission of locations’ names to English colons and their translation, as the dialect spoken by their aid also shaped the spelling. Hamilton’s book was used as an intermediary to identify, verify, and match modern locations, as the book contains coordinates that we could enter in Google earth. The coordinates are potentially inaccurate by today’s standard but, by looking at the vicinity of the indicated location, one could in most cases find the corresponding locality. Nevertheless, Jameson’s and Scot’s reports didn’t exclusively describe locations that were present in Hamilton’s index. For those locations, we had to obtain their locations by extracting the maximum of information about their vicinity from the original document, and using Google earth’s line function to triangulate their position. In parallel, we used a sufficiently detailed ancient map along with Google Map for localization.

Another specificity of modern locality names is their propensity to have homonyms. For all locations in this case, the location was determined using Google Maps, triangulating their position by entering all homonyms together with all other locations cited in the same paragraph in the source and selected homonym closest to other cited locations. When possible, the method was used as a last recourse to extrapolate the modern position of some localities for which we didn’t have a corresponding name in Hamilton’s book nor a trace on an ancient map. To locate villages on the ancient map, we used the parsing of the text as a reference coupled with active visual tracking on ancient maps accounting for topography and roads, together with all available descriptive material. From Scot’s and Jameson’s publications, we located around two hundred individual cities, towns and villages. Only ten remained unidentifiable. The correspondence work is provided in a separate file for future improvement, should new methods for interpretation of ancient location names into present administrative structures appear.

Once this coding work is realized, we obtain a first dataset, containing the presence and incidence of cholera for the whole Hindustan area, on a monthly basis.

More precisely, the following variables are present in the dataset:

- % Divisions with available data: provides information on the comparability of the data with Donaldson’s set. The coverage rate is defined as the ratio of the number of divisions with available data to the total number of divisions.
- M/A: “M” stands for “monthly” and “A” stands for “annually”.
- M/A coded: the above qualitative data is coded in the following way: 1 for “months”, 2 for “years”, 99 for “N/A”.
- MIL PREV: stands for “military prevalence”. This was calculated based on the number of cases per regiment. When the number of soldiers in a regiment was not communicated, the standard size of 1,000 soldiers per regiment was allocated. It presents a slight overestimation of the number of soldiers per regiments, thus, an underestimation of the prevalence.
- CIV PREV: stands for “civil prevalence”. The prevalence was compiled using the source when possible. For the localities present in the *Census of India 1961 Report on the population estimates of India (1820–1830)*, the prevalence could be calculated based on the number of deaths per 1,000 inhabitants.
- MIL PREV CODED/CIV PREV CODED: same definition as above, but the qualitative data is coded in the following way: 1 for “low”, 2 for “frequent”, 3 for “strong”, 4 for “exceptional” and 99 for “N/A”.
- Military cases: reported number of cholera cases among the military.
- Military deaths: reported number of deaths from cholera among the military.
- Military population: number of soldiers given by the reports, otherwise, 1,000 soldiers per regiment.
- Civil cases: reported number of cholera cases among the civilians.
- Civil deaths: reported number of deaths from cholera cases among the civilians.
- Civil population: based on the *Census of India 1961 Report on the population estimates of India (1820–1830)*.

Table 1 summarizes the information for this period, revealing, for example, that, although military prevalence is less documented than the civilian one (as, on average, we have information for 32 cells per year for the military part of the dataset, compared to 141 for the civilians), the number of deaths is on average higher among the military (almost 400 deaths per year, compared to 316 for the civilian population).

**Table 1:** Descriptive statistics, first period.

	1814–1824
% Divisions with Available Data	40.00
% Divisions with Available Data (Bangladesh)	66.00
% Divisions with Available Data (India)	38.00
% Divisions with Available Data (Nepal)	0.00
% Divisions with Available Data (Pakistan)	25.00
Months (average number of cells with monthly information)	139
Annual (average number of cells with yearly information)	18
MIL PREV (military prevalence, average number of cells with information)	32
CIV PREV (civilian prevalence, average number of cells with information)	141
Military Deaths	392.96
Civil Deaths	315.97

### Primary and secondary sources, period 1825–1905

The second part of the dataset is not as detailed as the first one, as it only accounts for the presence of cholera, but not for its incidence. Moreover, the information allows us to localize episodes of cholera for each year, instead of a monthly basis. As Table 2 reveals, the average number of divisions hit by a cholera episode is superior to 585, with a maximum of 2,172 in 1892. In the sample, India is much better covered than the other parts of the sub-continent.

**Table 2:** Descriptive statistics, second period.

	1825–1904
Hit by cholera	
Average number of divisions hit	585.29
Average number of divisions hit (Bangladesh)	52.00
Average number of divisions hit (India)	529.70
Average number of divisions hit (Nepal)	3.44
Average number of divisions hit (Pakistan)	0.15
Minimum (Year)	0 (1858)
Maximum (Year)	2,172 (1892)

**Fig. 2:**
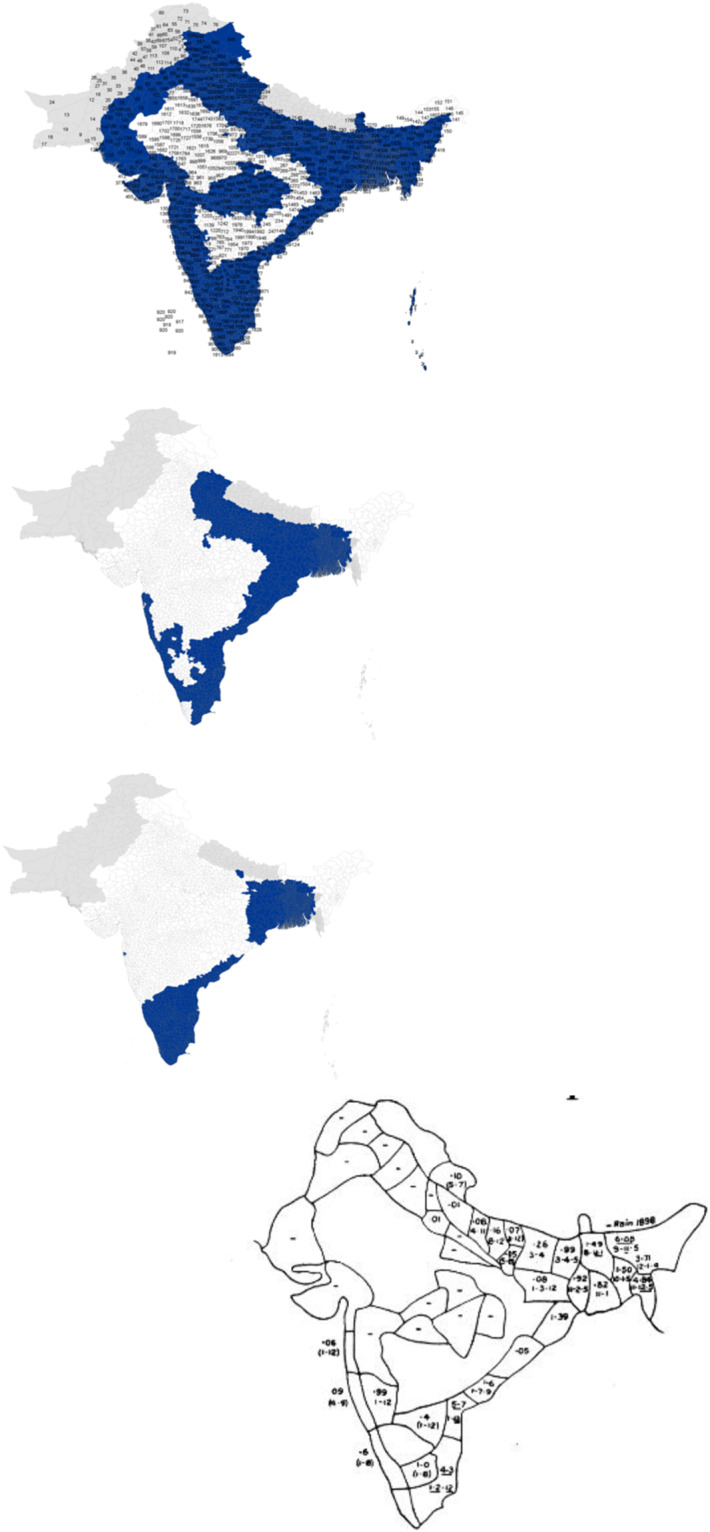
Example of comparison of data with ancient maps Rogers (1926, map for year 1898).

The methodology for this dataset was different than for the previous part. Most of it is based on the superposition of ancient maps treating the impact of cholera of Hindustan on a modern map of India, Pakistan, Nepal, and Bangladesh – see Fig. 2 for an example. For the rest, it relies on the parsing of medical articles and WHO reports on the disease – see Table 3.

**Table 3:** Second-period primary sources.

Date of burst of cholera	Source	Type	Medical consensus on source of epidemics
1814–1824	Jameson and Scot reports	Parsing	1817-21 Started in India
1825–1832	Brigham’s A treatise on epidemic cholera	Parsing	1829-51 Started in India
1855–1869	Bryden report and cholera in southern India	Maps	1852-60 Mainly in Russia
1866	Cholera In southern India	Parsing	1863–75 Started in Bengal
1875–1877	Report on the cholera epidemic of 1875 in India and The condition influencing the spread of cholera	Maps	1881-96 Started in India
1881	Pollitzer WHO report 1959 p38	Parsing (general description)	1899–1923 Started in India
1899–1904	Pollitzer WHO report 1959 p41	Parsing (general description)	1961–70 Started in Indonesia
1892	The condition influencing the spread of cholera	Parsing	
1898	The condition influencing the spread of cholera	Map	
1902	The Cholera Epidemic in Puri Town and District in July, 1902	Parsing	

Concerning the superposition method, firstly it has been relied on a modern map of India, Pakistan, Nepal, and Bangladesh, divided into divisions in ArcGIS. More precisely, the modern maps are divided in current districts/region/province, etc. They are superposed on the ancient map, and we check whether the new division is in an infected area or not. On top of this, an ancient map, adjusted for the differences in perspectives using Photoshop was used, and then we added the impacted divisions into the dataset.

When the division was only partially reliably similar by the outline of the ancient map, it wasn’t included in the dataset to favor underestimation of the presence of Cholera rather than overestimation. If a similar case happened but the division was in between two contaminated areas, it was included. The accuracy of each dataset was checked via visualization in ArcGIS and direct comparison with the source.

The areas studied here nowadays share a similar administrative structure, with a (modern) division in states, divided into districts, and then districts into divisions. The latter could be considered as a proxy for the most notable cities in a district plus their surroundings. The whole dataset has the granularity level of divisions.

## Data Records

The dataset^12^ carries an entry tag (identifier), time, and location information. The location is, when necessary, transformed from the “ancient” to the “modern” name, the former being the name as it appears in Hamilton’s book or the original source if not present in the book. Location “modern” is the name according to Google map in the dataset - it is as it appears in the GIS shapefile. We provide the coding used by Donaldson^4^ for further reference and for comparison purposes.

For the period 1814–1824, Population, Cases, Deaths variables were used to assess the severity of the epidemic for each segment. Prevalence is a categorical variable that indicates the severity of the disease based on the description of Scot^10^ and Jameson^9^. Prevalence can be Low, Frequent, Strong, or Exceptional. Categories were based on the calculation of military prevalence and also civilian prevalence when available, these two categories were differentiated and calculated separately. The measured importance of cholera is biased by Jameson and Scot’s point of view and their inclination to use reports from Medical Officers of the army; therefore, accounts of civilian prevalence are rare. When it comes to the description of the epidemic in civilian areas, they are generally accounting solely for deaths.

To determine the severity scale of cholera, we calculated military prevalence based on the number of cases per regiments. When the number of soldiers in a regiment was not communicated, we allocated a standard size of 1000 per regiment which is a slight overestimation of the number of soldiers per regiments, thus, a potential underestimation of the prevalence, to err on the safer side.

We then used the quartiles of the prevalence distribution to determine the severity. Observations in the first quartile are categorized as “Absent or very low”, they are “Frequent” in the second quartile, “Strong” in the third and “Exceptional” in the last one. Regiments were not the only type of military installations, these latter also cover Forts, Prisoners, Detachments. When the number of individuals in a detachment was not given, we allocated a value of 350 members, with a similar lower bias as for regiments. Table 4 details civilian and military prevalence quartiles expressed per 1/1000 people. Referring to Scot’s report, this method of categorizing prevalence fits with the historical reality of marginally exceptional prevalence levels in the military troops, as he considered 270/1000 and 380/1000 to be exceptional levels.

**Table 4:** Coding prevalence (per 1000).

	Min	Quartile 1	Quartile 2	Quartile 3	Max
Civilian	0	14	27	89	168
Military	0	17	27	134	471

For civilian prevalence, we compiled the data using the original source when possible. For extrapolation of prevalence, there was the problem that civilian localities can’t be allocated a fixed number of inhabitants. For the localities whose name is present in a given report on population estimates in India between 1820 to 1830, we could estimate the prevalence, while it was not possible before.

In the end, the dataset categorizes prevalence to be strong above 27/1000 and exceptional above 134/1000. In the cases where Scot reported an episode of epidemic cholera but didn’t include a description of the severity, we assigned the grade Low to the event, still to be on a safe side of underestimation.

## Technical Validation

The detective work involved in creating the dataset pertaining to 1814–1824 is both a strength and a limitation of this dataset. On some occasions, a wrong location - due to the imperfect information and difficulty of tracking localities through history - may have been recorded. Problems that might cause inaccuracies are locations shifting geographically, that is to say, the town subtly changed its name, and the ancient name is borne by another location nearby in modern times.

**Fig. 3:**
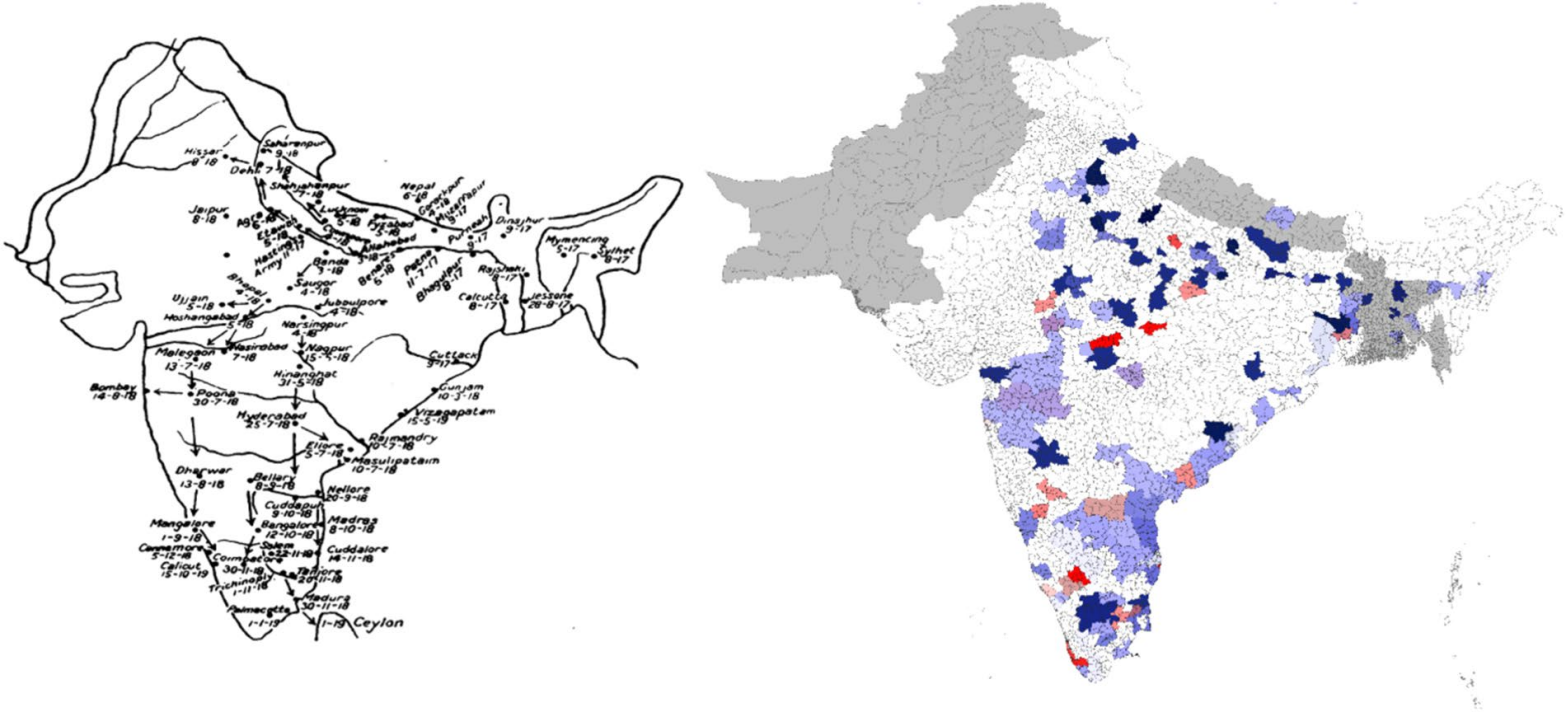
Example of comparison of data with ancient maps. Rogers (1926, map for 1817–1819).

Another problem is the description of localities in two sources with different spelling and homonym locations with spelling variations in close vicinity. In these examples, the use of ancient maps with roads helped greatly in avoiding such discrepancies, but they are telling of the level of subjectivity necessary to reach a fine granularity of geocoding. Subjectivity induced in the dataset might distort facts. Nonetheless, the comparison in Fig. 3 provides a robustness check, confirming that this dataset is generally correct.

Regarding the first period, we argue that Scot’s and Jameson’s reports, on which the first part of the dataset is strongly based, are key references about the episodes of cholera between 1814 and 1824 in the whole Hindustan. When searching for corresponding data on the location, most were cited by these primary sources. A map displaying the cholera epidemics of 1817 to 1819 found after parsing and plotting, seems to corroborate the geocoding of Scot’s and Jameson’s report - see Fig. 3.

## Usage Note

An obvious limitation is that the coverage is not complete through time and space. For a given year, some localities are either not covered (there are no data) while others have a rate of coverage going from 7.7% to 100%. It means that for each geographical entity having an observation, the data we provide may come from only 7.7% of the localities included in the entity to 100%. Any future use of the dataset should take this into account, potentially by using the variable “% Divisions with Available Data” as an indicator of the level of imputation. Any robustness checks should probably exclude localities with the lowest rates of coverage. For the first part of our dataset, covering the period 1814–1904, any future user should also consider coverages indicated in Tables 1, 2 as a useful indicator of the level of representativeness of our measures by year and country.

Despite this limitation, in our view, the dataset could be used to bring new lights on existing works. For example, the analysis of the productivity of Indian railways between 1874 and 1912 (Bogart and Chaudary^13^) could be differentiated by the geographic prevalence of the epidemic. It could also be the case that complementary datasets may bring light into the impact of epidemics in the long-run, and that cholera may be as important as the plague (Siuda and Sunde^14^). The historical presence of Cholera as we document it could also help understanding the roots of cultural persistence in India, regarding for instance the status of women^15^ and beyond^16^. Overall, this data will be valuable for studies of the long-term effects of epidemics in different contexts^12^.

## Data Availability

No particular code was created for this dataset.
